# In-silico testing of new pharmacology for restoring inhibition and human cortical function in depression

**DOI:** 10.1038/s42003-024-05907-1

**Published:** 2024-02-23

**Authors:** Alexandre Guet-McCreight, Homeira Moradi Chameh, Frank Mazza, Thomas D. Prevot, Taufik A. Valiante, Etienne Sibille, Etay Hay

**Affiliations:** 1 Krembil Centre for Neuroinformatics, Centre for Addiction and Mental Health, Toronto, ON Canada; 2 Krembil Brain Institute, University Health Network, Toronto, ON Canada; 3 Department of Physiology, University of Toronto, Toronto, ON Canada; 4 Department of Psychiatry, University of Toronto, Toronto, ON Canada; 5 Campbell Family Mental Health Research Institute, Centre for Addiction and Mental Health, Toronto, ON Canada; 6 Institute of Medical Sciences, University of Toronto, Toronto, ON Canada; 7 Department of Electrical and Computer Engineering, University of Toronto, Toronto, ON Canada; 8 Institute of Biomaterials and Biomedical Engineering, University of Toronto, Toronto, ON Canada; 9 Department of Surgery, University of Toronto, Toronto, ON Canada; 10 Center for Advancing Neurotechnological Innovation to Application, Toronto, ON Canada; 11 Max Planck-University of Toronto Center for Neural Science and Technology, Toronto, ON Canada; 12 Department of Pharmacology and Toxicology, University of Toronto, Toronto, ON Canada

**Keywords:** Biophysical models, Depression, Neural circuits

## Abstract

Reduced inhibition by somatostatin-expressing interneurons is associated with depression. Administration of positive allosteric modulators of α5 subunit-containing GABA_A_ receptor (α5-PAM) that selectively target this lost inhibition exhibit antidepressant and pro-cognitive effects in rodent models of chronic stress. However, the functional effects of α5-PAM on the human brain in vivo are unknown, and currently cannot be assessed experimentally. We modeled the effects of α5-PAM on tonic inhibition as measured in human neurons, and tested in silico α5-PAM effects on detailed models of human cortical microcircuits in health and depression. We found that α5-PAM effectively recovered impaired cortical processing as quantified by stimulus detection metrics, and also recovered the power spectral density profile of the microcircuit EEG signals. We performed an α5-PAM dose-response and identified simulated EEG biomarker candidates. Our results serve to de-risk and facilitate α5-PAM translation and provide biomarkers in non-invasive brain signals for monitoring target engagement and drug efficacy.

## Abstract (web summary)

Using data-driven models of human neurons and microcircuits, the authors tested in-silico a compound for treating depression via boosting cell-specific inhibition, and identified EEG biomarker candidates for monitoring treatment efficacy.

## Introduction

A loss of cortical inhibition is associated with major depressive disorder (depression)^1^, and studies indicate the involvement of somatostatin-expressing (SST) inhibitory interneurons^2–11^. Functionally, cortical SST interneurons mediate lateral inhibition through inhibitory disynaptic loops^12,13^ and provide a “blanket of inhibition” that maintains low Pyr neuron spike rates at baseline^14–16^. A reduced inhibition due to reduced SST expression in SST interneurons in depression^10^ is supported by studies showing that the SST peptide is co-released with GABA^17^, SST receptor activation directly elicits inhibitory responses in cortical pyramidal neurons, even while GABA receptors are blocked^18^, and SST administration can elicit anti-depressant effects^19^. SST interneurons primarily target the apical dendrites of pyramidal (Pyr) neurons, where they provide both synaptic and extrasynaptic (i.e., tonic) inhibition via the α5 subunit of GABA_A_ (α5-GABA_A_) receptors^20–23^. While mostly studied in rodents, studies showed that α5-GABA_A_ is similarly expressed in human cortical pyramidal neurons and negligibly in interneurons^24^. Accordingly, tonic inhibitory currents generated by α5-GABA_A_ receptors have been recorded in human cortical pyramidal neurons and are mostly absent in interneurons^25^.

Novel benzodiazepine-like compounds with preferential affinities and positive allosteric modulation of α5-GABA_A_ receptors (α5-PAM) have been shown to elicit anxiolytic, antidepressant, and pro-cognitive effects in rodent models associated with reduced dendritic inhibition, such as chronic stress or aging, and are therefore promising new treatments for depression^26–31^. Comparatively, non-selective benzodiazepines (i.e., targeting a broader range of GABA_A_ receptor subunits)^32^ that are efficacious at treating anxiety^33^ have several undesirable side-effects that include sedation, ataxia, amnesia, and abuse liability due to their broad modulation of cortical inhibition via α1 subunit-containing GABA_A_ receptors^34,35^, which are expressed more ubiquitously across neuron types^36,37^. While α5-PAM effects on rodents suggest a potential role in the treatment of depression, their effects on human brain microcircuits remain unknown due to experimental and ethical limitations, thus meriting in silico testing.

There are two main factors that make testing α5-PAM effects on human microcircuits not trivial. First, although there are many similarities between cortical microcircuits in humans and other species, there are also important differences. Compared to rodents, human cortical circuits exhibit stronger excitatory and inhibitory synaptic connections^12,38–41^, as well as larger morphological sizes and enhanced dendritic compartmentalization^42–47^. The effects of α5-PAM in rodents are therefore expected to translate to humans, but the extent to which species differences modify these effects remains to be determined. Another non-trivial factor in testing α5-PAM effects is the difference between the α5-PAM mode of operation and the underlying SST interneuron mechanisms of depression. α5-PAM in cortex acts specifically to enhance inhibition onto Pyr neuron apical dendrites^20–23^, whereas reduced SST interneuron inhibition affects additional microcircuit connections from SST interneurons onto other interneuron subtypes^22,48^. Thus, it is unclear whether α5-PAM is sufficient to fully recover circuit activity dynamics following reduced SST interneuron inhibition in depression.

Here we test in silico the effects of α5-PAM (GL-II-73)^31^ on human microcircuit spiking, function in terms of signal detection, and EEG signals, using our previous detailed models of human cortical microcircuits in health and depression^48^. We characterize the effects of different doses of α5-PAM, identify biomarker candidates in EEG signals and establish their specificity compared to non-selective GABA_A_ receptor PAMs.

## Results

### Data-driven models of α5-PAM modulation in human neurons and in-silico testing of microcircuit function recovery

**Fig. 1:**
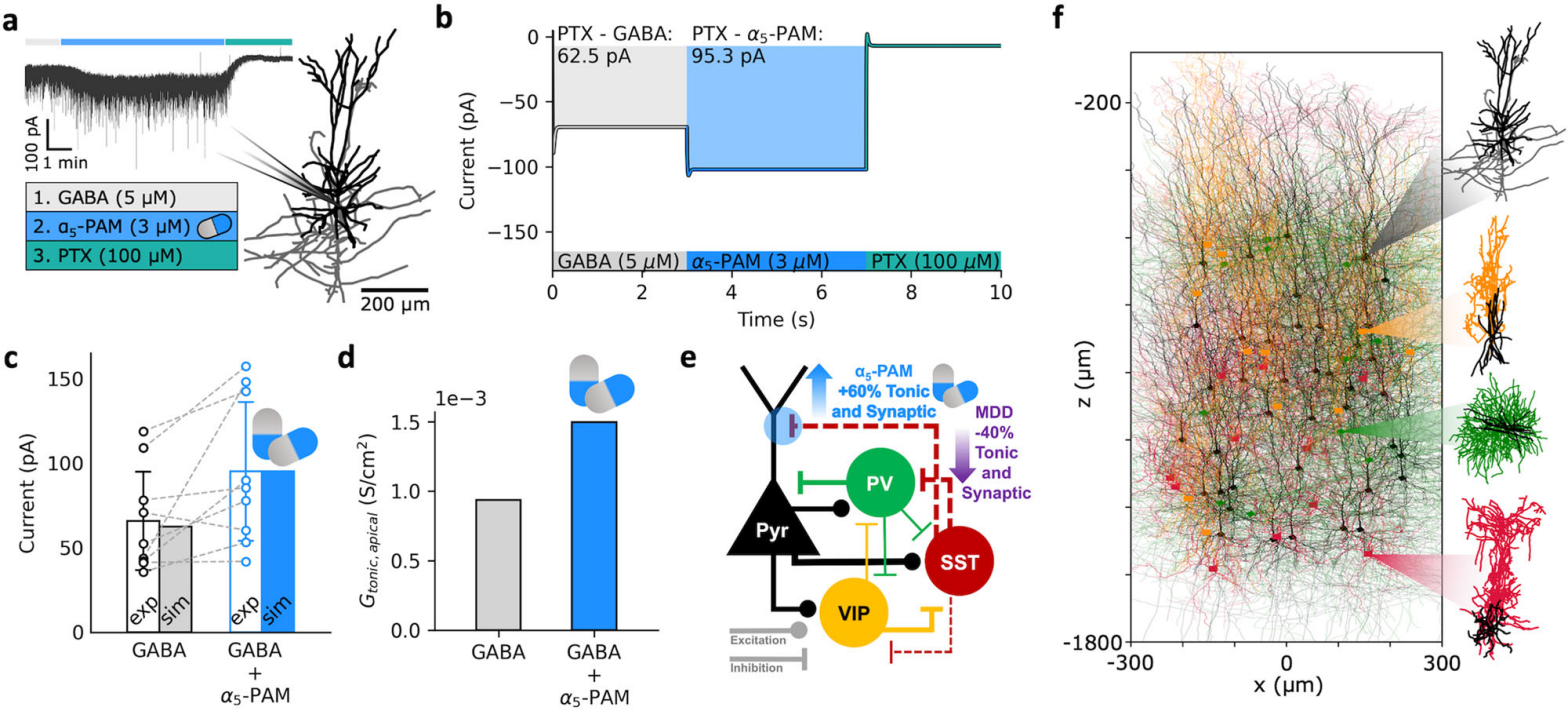
Data-driven simulation of α5-PAM effect on human neurons and microcircuits. **a** Illustration of the detailed human L2/3 pyr neuron model used in the study and an example voltage-clamp recording under the different experimental conditions (GABA: application of GABA, α5-PAM: application of α5-PAM + GABA, PTX: GABA block). **b** Simulated tonic inhibition current recordings at soma in the different conditions, fitted to reproduce the experimentally recorded current magnitude averages. **c** Comparison of experimental (error bars show SD) and simulated current output magnitudes (relative to the output magnitude in the PTX condition) in the GABA (gray) and α5-PAM + GABA (blue) conditions. **d** Derived apical tonic inhibition conductance values in the GABA (gray) and α5-PAM + GABA (blue) conditions. **e** Schematic of the model L2/3 microcircuit connectivity and summary of depression (MDD) and derived α5-PAM effects on the microcircuit. **f** Illustration of the detailed L2/3 microcircuit models with human model morphologies (from top to bottom: Pyr, VIP, PV, SST, color-coded as in e).

We first experimentally recorded tonic inhibition currents in single healthy human pyramidal neurons in the presence of GABA only, and in the presence of α5-PAM + GABA, and found a 52% increase in current magnitude during application of a reference dose of α5-PAM (3 µM) in addition to GABA (-65.9 ± 29.08 pA vs -95.3 ± 41.08 pA, one-sided paired-sample t-test, *p* = 0.02, Cohen’s *d* = 0.82; Fig. 1a, c). We then modeled the α5-PAM modulation in single neurons by constraining our previous human L2/3 Pyr neuron model to reproduce the average currents recorded in-vitro (Fig. 1b, c). We first fitted the tonic inhibition conductance (*G*_*tonic*_) in the neuron (uniformly in soma, basal dendrites, and apical dendrites) to reproduce the current magnitude recorded when applying GABA, and then fitted the α5-PAM modulation of *G*_*tonic*_ in the apical dendrites to reproduce the 52% increase in tonic current magnitude. We thus estimated the α5-PAM modulation to be a 60% increase in conductance (Fig. 1d).

We next integrated this modulation into our previous biophysically detailed models of human L2/3 microcircuits in health and depression^48^. The microcircuit models included key neuron types (Pyr, PV, SST, and VIP neurons), and the depression microcircuits involved a 40% reduction in SST interneuron synaptic and tonic inhibition onto the other neurons (Fig. 1e, f). We also applied the estimated α5-PAM modulation to synaptic inhibition mediated by SST interneurons onto Pyr neuron apical dendrites, since these connection types in the cortex are α5-mediated.

**Fig. 2:**
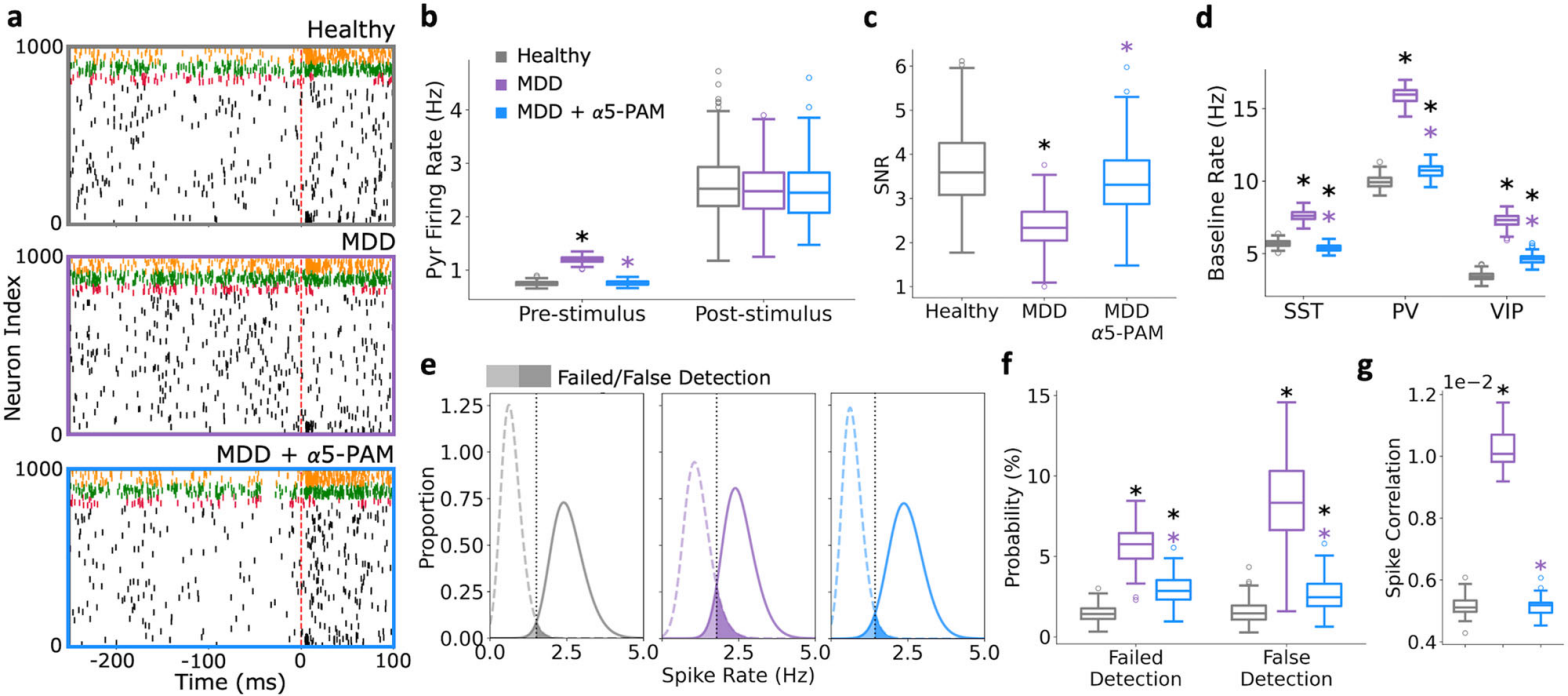
In silico application of α5-PAM in depression microcircuits restores healthy spike rates and function. **a** Example raster plots of simulated baseline spiking and response to brief stimulus in healthy (top), depression (middle), and depression + α5-PAM (bottom) microcircuit models. The dashed line indicates stimulus time. Cell type color code is the same as in Fig. 1e-f. **b** Pre- and post-stimulus Pyr neuron firing rates in the different simulated conditions. α5-PAM restores healthy levels of pre-stimulus firing. **c** SNR of response in each condition, where α5-PAM boosts SNR to healthy levels. **d** Baseline interneuron firing rates in the different simulated conditions. **e** Distributions of pre- and post-stimulus firing rates. The vertical lines denote the decision boundaries, and the shaded areas show the failed/false detections. **f** Probability of failed detection and false detection in each simulated condition. α5-PAM significantly reduced failed/false detection rates, bringing them close to healthy levels. **g** Mean pairwise spike correlations across all Pyr neurons in each condition (spikes binned into 0-1 spike train vectors, bin size = 1 ms). All asterisks denote significant paired t-tests (*p* < 0.05) with effect sizes greater than 1, when compared to healthy (black asterisks) or depression (MDD; purple asterisks). n = 200 randomized microcircuit simulations per condition for panels **b**–**f** and 20 randomized microcircuit simulations per condition for panel **g**. For all box-and-whisker plots, the boxes show the interquartile range, the middle lines show the medians, the whiskers extend to all data within 1.5x the IQR, and the dots show data points outside of the whisker range.

We next tested the α5-PAM effects on the human cortical microcircuits by simulating microcircuit baseline and response to brief stimuli in health, depression, and depression + α5-PAM (Fig. 2). As we showed previously, compared to healthy circuits, reduced SST interneuron inhibition in depression microcircuits resulted in increased baseline spike rates (Fig. 2a, b; healthy: 0.75 ± 0.04 Hz; depression: 1.20 ± 0.06 Hz; paired-sample t-test, *p* = 5.52e−204, Cohen’s *d* = 8.6), decreased SNR (Fig. 2c; healthy: 3.73 ± 0.91; depression: 2.37 ± 0.49; paired sample *t*-test, *p* = 2.03e−51, Cohen’s *d* = −1.9), and worsened microcircuit function in terms of failed and false stimulus detection rates, calculated based on the distribution of Pyr neuron firing at baseline vs response averaged over 50 ms windows (Fig. 2e, f; healthy: 1.45 ± 0.47% and 1.54 ± 0.63%, depression: 5.71 ± 1.10% and 8.44 ± 2.53%, paired-sample *t*-test, *p* = 6.54e−125 and 2.32e−96, Cohen’s *d* = 5.0 and 3.7 respectively). Modulation of inhibition by a reference dose of α5-PAM in simulated depression microcircuits restored baseline spike rates (Fig. 2b; 0.76 ± 0.04 Hz, Cohen’s *d* = 0.2) and consequently SNR (Fig. 2c; 3.42 ± 0.80, Cohen’s *d* = −0.4) back to healthy levels. In both the depression and α5-PAM conditions, there were no effects on post-stimulus firing rate (Fig. 2b). Similar effects on firing rates across conditions were seen with the SST, PV, and VIP interneuron populations (increased rates in depression vs healthy, Cohen’s *d*: SST = 6.7, PV = 12.7, VIP = 10.5; recovery by α5-PAM vs healthy, Cohen’s *d*: SST = −1.4, PV = 1.8, VIP = 4.0; Fig. 2d). The simulated reference dose of α5-PAM also recovered the failed and false stimulus detection rates nearly back to healthy level (Fig. 2e-f**;** 2.91 ± 0.83% and 2.59 ± 0.97%, Cohen’s *d* = 2.2 and 1.3). Depression microcircuits had increased spike correlations between Pyr neurons at baseline (healthy: 0.0052 ± 0.0004, depression: 0.0103 ± 0.0007, *p* = 1.62e−17, Cohen’s *d* = 8.8), which was recovered back to healthy levels after application of α5-PAMs (0.0052 ± 0.0004, Cohen’s *d* = 0.01; Fig. 2g).

**Fig. 3:**
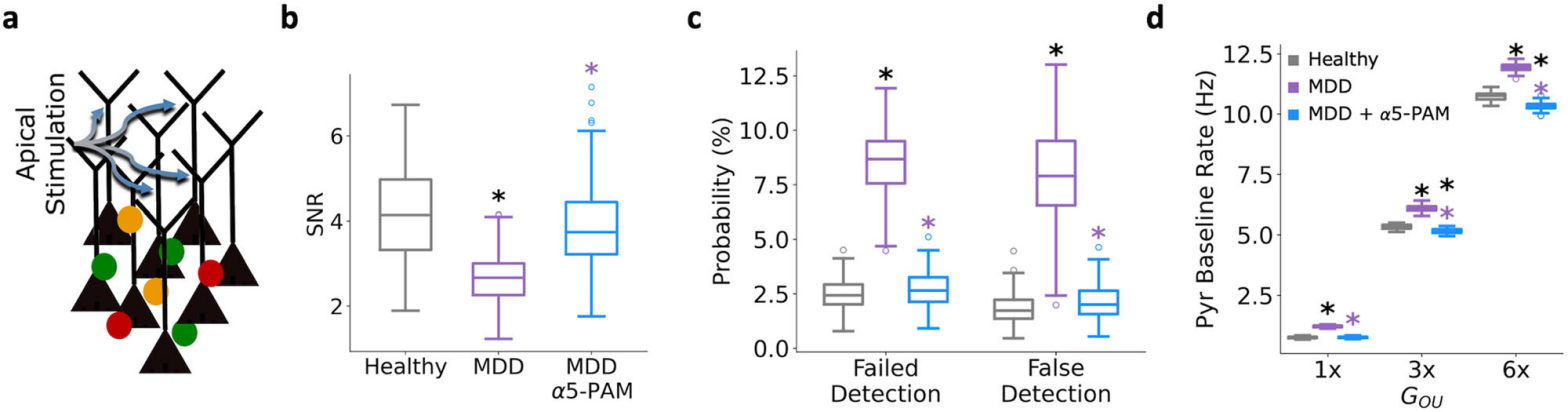
In-silico application of α5-PAM in depression microcircuits restores apical dendritic processing and a range of activity states. **a** Schematic of apical dendritic stimulation. **b** Microcircuit spiking SNR in each condition. **c** Probability of failed detection and false detection in each simulated condition. **d** Averaged baseline Pyr neuron firing rates in each condition and background input level. All asterisks denote significant paired *t*-tests (*p* < 0.05) with effect sizes greater than 1, when compared to healthy (black asterisks) or depression (MDD; purple asterisks). *n* = 200 randomized microcircuit simulations per condition for panels **a**–**c** and 50 randomized microcircuit simulations per condition for panel **d**. For all box-and-whisker plots, the boxes show the interquartile range, the middle lines show the medians, the whiskers extend to all data within 1.5x the IQR, and the dots show data points outside of the whisker range.

We also examined recovery of apical dendritic processing, by applying the stimulus to Pyr neuron apical dendrites instead of basal dendrites (Fig. 3a). We found that α5-PAM recovered SNR (healthy vs α5-PAM Cohen’s *d* = −0.3, Fig. 3b), and even better recovered failed and false detection rates based on apical inputs (healthy vs α5-PAM Cohen’s *d*: failed = 0.3, false = 0.4, Fig. 3c) compared to basal input processing. In addition, we probed the ability of α5-PAM to recover middle and high levels of baseline Pyr neuron rates, by simulating a baseline firing rate of ~5 Hz (3x *G*_*OU*_ background input to Pyr neurons) and ~10 Hz (6x *G*_*OU*_), respectively (Fig. 3d). We found that even in these higher activity states α5-PAMs recovered the firing rate back close to healthy levels (depression vs α5-PAMs – Cohen’s *d*: 1x *G*_*ou*_ = −10.5; 3x *G*_*ou*_ = −7.8; 6x *G*_*ou*_ = −9.4; healthy vs α5-PAMs – Cohen’s *d*: 1x *G*_*ou*_ = 0.2; 3x *G*_*ou*_ = −1.6; 6x *G*_*ou*_ = −2.4; Fig. 3d).

**Fig. 4:**
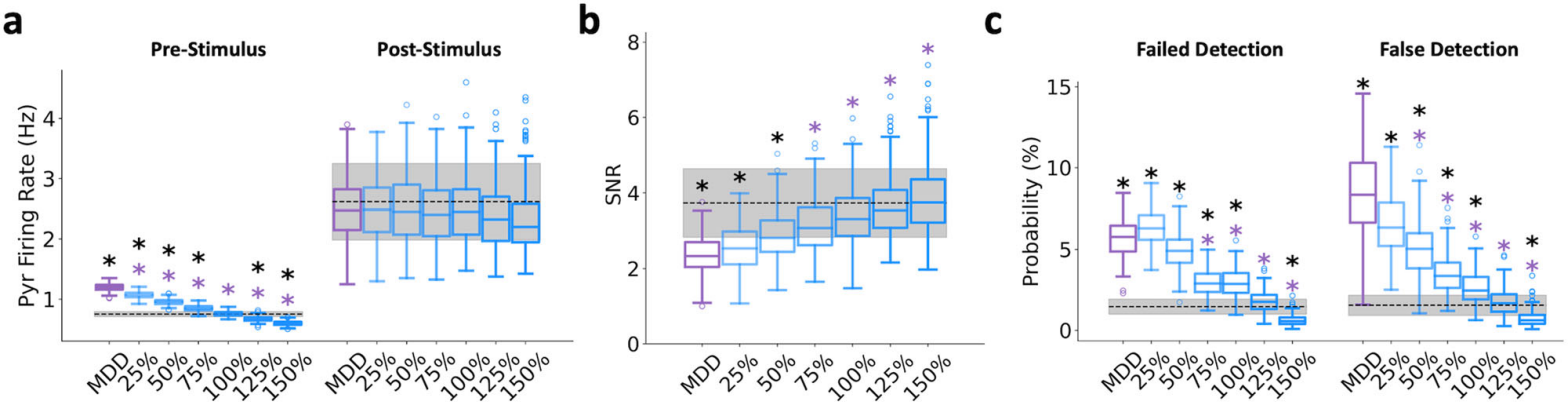
In silico dose-response highlights optimal levels for maximizing drug effects. Pyr neuron pre- and post-stimulus firing rates (**a**), SNR (**b**), and failed and false detection (**c**) for depression (MDD; purple) and different doses of α5-PAM (shades of blue: 25–150% of the estimated reference dose of α5-PAM). The black horizontal lines and shaded areas denote the healthy mean ± standard deviation. All asterisks denote significant paired t-tests (*p* < 0.05) with effect sizes greater than 1, when compared to healthy (black asterisks) or depression (MDD; purple asterisks). *n* = 200 randomized microcircuit simulations per condition. For all box-and-whisker plots, the boxes show the interquartile range, the middle lines show the medians, the whiskers extend to all data within 1.5x the IQR, and the dots show data points outside of the whisker range.

To test the effectiveness of higher or lower α5-PAM doses than the reference dose at recovering microcircuit function, we simulated α5-PAM modulation of apical inhibition ranging from 25–150% of the estimated modulation by the experimental reference dose (Fig. 4). The reference dose of α5-PAM (referred to as 100%) turned out to be the optimal dose at recovering baseline spike rates back to healthy levels in the simulated microcircuits (healthy: 0.75 ± 0.04 Hz; 100% α5-PAM: 0.76 ± 0.04 Hz, Cohen’s *d* = 0.2), whereas lower doses were not sufficient (25% α5-PAM: 1.07 ± 0.05 Hz, paired-sample t-test, *p* = 2.49e−190, Cohen’s *d* = 6.5) and higher doses over-reduced the spike rate (150% α5-PAM: 0.60 ± 0.04 Hz, paired-sample t-test, *p* = 2.69e−146, Cohen’s *d* = −3.8; Fig. 4a). The relationship between dose and effect on baseline rates was linear (Pearson correlation, r = −0.97, *p* = 0.0) and was only marginally better fitted by exponential or sigmoidal fits ( ~ 10% improvement in the sum of squared errors). None of the doses tested had effects on post-stimulus firing rate (Fig. 4a). The relationship between dose and SNR was also linear (Pearson correlation, r = 0.58, *p* = 1.35e−126; <5% difference in sum of squared errors between linear, sigmoid and exponential fits), with several doses restoring SNR back to healthy levels (100–150%; Fig. 4b; healthy: 3.73 ± 0.91; 100% α5-PAM: 3.42 ± 0.80, Cohen’s *d* = −0.4; 125% α5-PAM: 3.66 ± 0.87, Cohen’s *d* = −0.1; 150% α5-PAM: 3.92 ± 0.99, Cohen’s *d* = 0.2). We note, however, that for doses greater than 100% the SNR was preserved because both baseline and response rates were similarly dampened, whereas the 100% dose effect involved minimal dampening and thus was more optimal. The relationship between dose and failed/false stimulus detection errors was nearly linear (Pearson correlation, r = −0.88 and −0.85, respectively, *p* = 0.0 and 0.0; <10% difference in the sum of squared errors between linear, exponential and sigmoid fits), with a poor effect for low doses (25–50%; 25% α5-PAM: 6.36 ± 1.09% and 6.57 ± 1.95%, paired-sample *t*-test, *p* = 3.79e−141 and 1.02e−93, Cohen’s *d* = 5.8 and 3.5, compared to healthy), and errors rates recovering close to healthy levels for doses of 75–125% (Fig. 4c; 100% α5-PAM: 2.91 ± 0.83% and 2.59 ± 0.97%, paired-sample *t*-test, *p* = 6.83e−65 and 5.94e−36, Cohen’s *d* = 2.2 and 1.3, compared to healthy). The highest dose (150%) further reduced the failed and false error rates even below the healthy level (150% α5-PAM: 0.61 ± 0.32% and 0.73 ± 0.48%, paired-sample *t*-test, *p* = 2.01e−60 and 6.15e−40, Cohen’s *d* = −2.1 and −1.4, compared to healthy), possibly through a greater dampening of pre-stimulus activity compared to post-stimulus when compared to healthy (Cohen’s *d* = −3.8 and −0.5, respectively).

### In-silico EEG biomarkers of α5-PAM efficacy in depression microcircuits

**Fig. 5:**
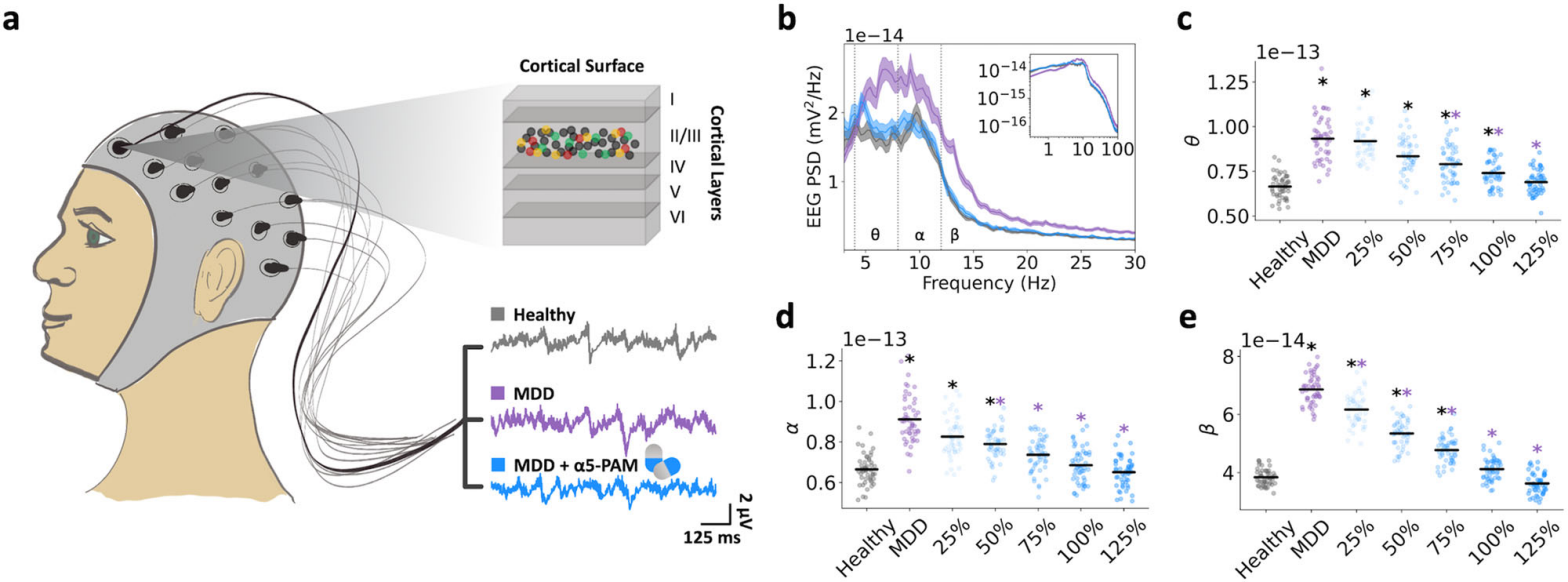
EEG power spectral biomarkers of α5-PAM efficacy. **a** Illustration of EEG signals generated from the human cortical microcircuit models. **b** Power spectral density (PSD) of simulated EEG from microcircuit models in each condition as labeled in a (bootstrapped mean, and 95% confidence intervals). α5-PAM restored the PSD profile to healthy level. Inset – PSD plotted in log scale. PSD in theta (4–8 Hz, **c**), alpha (8–12 Hz, **d**), and beta (12–21 Hz, **e**) bands for different α5-PAM doses (as in Fig. 4). All asterisks denote significant paired *t*-tests (*p* < 0.05) with effect sizes greater than 1, when compared to healthy (black asterisks) or depression (MDD; purple asterisks). *n* = 50 randomized microcircuit simulations per condition. Head picture in panel **a**: courtesy of A. Sherrington.

We simulated EEG together with the microcircuit activity in health and depression conditions to identify signatures of α5-PAM efficacy in a clinically-relevant non-invasive signal (Fig. 5a). As we previously showed^49^, simulated EEG signals generated by the depression microcircuit models with reduced SST interneuron inhibition exhibited increased power in theta (healthy: 6.65 × 10^−14^ ± 6.34 × 10^−15^ mV^2^; depression: 9.34 × 10^−14^ ± 1.20 × 10^−14^ mV^2^; paired-sample *t*-test, *p* = 6.15e−40, Cohen’s *d* = 2.8), alpha (healthy: 6.65 × 10^−14^ ± 7.32 × 10^−15^ mV^2^; depression: 9.12 × 10^−14^ ± 1.15 × 10^−14^ mV^2^; paired-sample t-test, *p* = 4.64e−17, Cohen’s *d* = 2.5), and beta (Healthy: 3.84 × 10^−14^ ± 2.78 × 10^−15^ mV^2^; depression: 6.86 × 10^−14^ ± 4.99 × 10^−15^ mV^2^; paired-sample t-test, *p* = 3.43e−41, Cohen’s *d* = 7.4) frequency bands (Fig. 5b). When α5-PAM was applied to the microcircuits at the reference dose, the power spectral density profile was restored close to the healthy at all frequency bands, except for a slight shift in theta band peak (Fig. 5b; 100% α5-PAM compared to healthy - θ: 7.41 × 10^−14^ ± 7.71 × 10^−15^ mV^2^, Cohen’s *d* = 1.1; α: 6.85 × 10^−14^ ± 8.44 × 10^−15^ mV^2^, Cohen’s *d* = 0.2; β: 4.12 × 10^−14^ ± 3.78 × 10^−15^ mV^2^, Cohen’s *d* = 0.8). There was a linear relationship between the effect of different α5-PAM doses at restoring power in theta (Fig. 5c; Pearson correlation, r = −0.67, *p* = 3.26e−41; < 1% difference in the sum of squared errors between linear, exponential and sigmoid fits), alpha (Fig. 5d; Pearson correlation, r = −0.70, *p* = 3.10e−45; < 1% difference in the sum of squared errors between linear, exponential and sigmoid fits), and beta (Fig. 5e; Pearson correlation, r = −0.92, *p* = 8.95e−127; <5% difference in the sum of squared errors between linear, exponential and sigmoid fits) frequency bands. The reference dose restored the power profile in alpha and beta bands (Fig. 5d, e), and a higher dose was required to restore the power profile in the theta band (Fig. 5c; 125% α5-PAM compared to healthy - θ: 6.90 × 10^−14^ ± 6.60 × 10^−15^ mV^2^, Cohen’s *d* = 0.4).

**Fig. 6:**
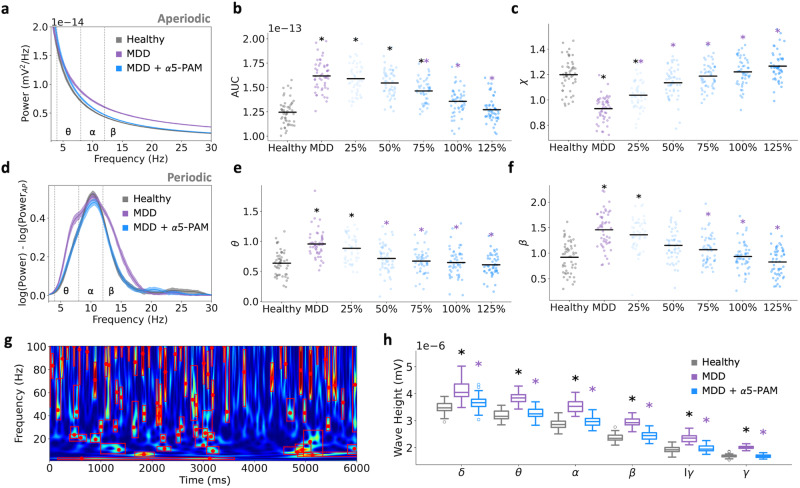
Decomposed EEG power spectral and oscillatory event biomarkers of α5-PAM efficacy. **a** Aperiodic components of the PSD for each condition (healthy: black; depression: purple; depression + 100% α5-PAM: blue). Broadband power spectral density area under the curve (AUC, 3–30 Hz, **b**) and exponent (χ, **c**) of the aperiodic component of the PSD for each α5-PAM dose (100%: fitted parameters for the 3 μM experimental α5-PAM dose). **d** Periodic component of the PSD for each condition (healthy: black; depression: purple; depression + 100% α5-PAM: blue). Power of the periodic components of PSD in theta (θ, 4-8 Hz; **e**) and beta (β, 12-21 Hz; **f**) bands for each α5-PAM dose. **g** Example simulated spectrogram of a healthy microcircuit, showing detection of oscillation events. The red boxes highlight the frequency spreads and durations of the events. The red dots indicate the peak frequencies. **h** Wave height extracted from oscillation events and separated by frequency bands. All asterisks denote significant paired *t*-tests (*p* < 0.05) with effect sizes greater than 1, when compared to healthy (black asterisks) or depression (MDD; purple asterisks). *n* = 50 randomized microcircuit simulations per condition. For all box-and-whisker plots, the boxes show the interquartile range, the middle lines show the medians, the whiskers extend to all data within 1.5x the IQR, and the dots show data points outside of the whisker range.

To further assess features of the spectral biomarkers of α5-PAM efficacy, we decomposed the power spectral density profiles into aperiodic (Fig. 6a–c) and periodic (Fig. 6d–f) components. In the periodic component of healthy microcircuits, we identified theta peaks in 64% of microcircuit simulations, alpha peaks in 100%, and beta peaks in 60%, and these numbers of identified peaks were not significantly altered across conditions. The lower portion of theta and beta peak resulted in them being less visible in the averaged PSD. As we have shown previously^49^, depression microcircuits with reduced SST interneuron inhibition primarily exhibited altered aperiodic exponents (healthy: 1.20 ± 0.13 mV^2^ Hz^−1^; depression: 0.93 ± 0.10 mV^2^ Hz^−1^; paired-sample t-test, *p* = 4.98e−16, Cohen’s *d* = −2.3) and broadband power (healthy: 1.24 × 10^−13^ ± 1.29 × 10^−14^ mV^2^; depression: 1.62 × 10^−13^ ± 1.53 × 10^−14^ mV^2^; paired-sample t-test, *p* = 7.67e−20, Cohen’s *d* = 2.6) as well as increased periodic theta frequency power (healthy: 0.64 ± 0.20 mV^2^; depression: 0.96 ± 0.22 mV^2^; paired-sample *t*-test, *p* = 3.39e-11, Cohen’s *d* = 1.5) and increased low-beta frequency power (healthy: 0.92 ± 0.30 mV^2^; depression: 1.46 ± 0.35 mV^2^; paired-sample *t*-test, *p* = 1.03e−10, Cohen’s *d* = 1.6, Fig. 6). The reference α5-PAM dose (100%) restored the aperiodic power and exponent back to healthy level (100% α5-PAM, power: 1.36 × 10^−13^ ± 1.48 × 10^−14^ mV^2^, Cohen’s *d* = 0.8; exponent: 1.22 ± 0.11 mV^2^ Hz^−1^, Cohen’s *d* = 0.2 compared to healthy, Fig. 6a–c), as well as the periodic power in theta and alpha bands (100% α5-PAM, θ: 0.65 ± 0.23 mV^2^, Cohen’s *d* = 0.05; β: 0.93 ± 0.27 mV^2^, Cohen’s *d* = 0.05 compared to healthy, Fig. 6d–f). The relationship between α5-PAM dose and the effect on aperiodic or periodic components was linear (Pearson correlation, aperiodic power: r = −0.65, *p* = 5.96e−37, exponent: r = 0.71, *p* = 4.81e−47, θ: r = −0.48, *p* = 1.04e−18, β: r = −0.59, *p* = 5.45e−30; <6% difference in the sum of squared errors between linear, exponential, and sigmoid fits).

As an additional check we performed a wavelet-based spectrogram analysis to identify individual oscillatory events (Fig. 6g, h), and found many oscillatory events across all frequency bands (δ, θ, α, β, lower γ, γ; Fig. 6g) in healthy and depression microcircuits. EEG changes in depression involved a robust increase in the event wave height across all frequency bands (healthy vs depression Cohen’s *d*: δ = 2.2, θ = 3.4, α = 3.3, β = 3.9, lower γ = 3.1, γ = 4.8), which was recovered with α5-PAM (healthy vs α5-PAM Cohen’s *d*: δ = 0.7, θ = 0.4, α = 0.5, β = 0.4, lower γ = 0.3, γ = 0.02; Fig. 6h). All other the event features (cycle count, event duration, peak frequency, frequency span, event count) remained unchanged across conditions.

**Fig. 7:**
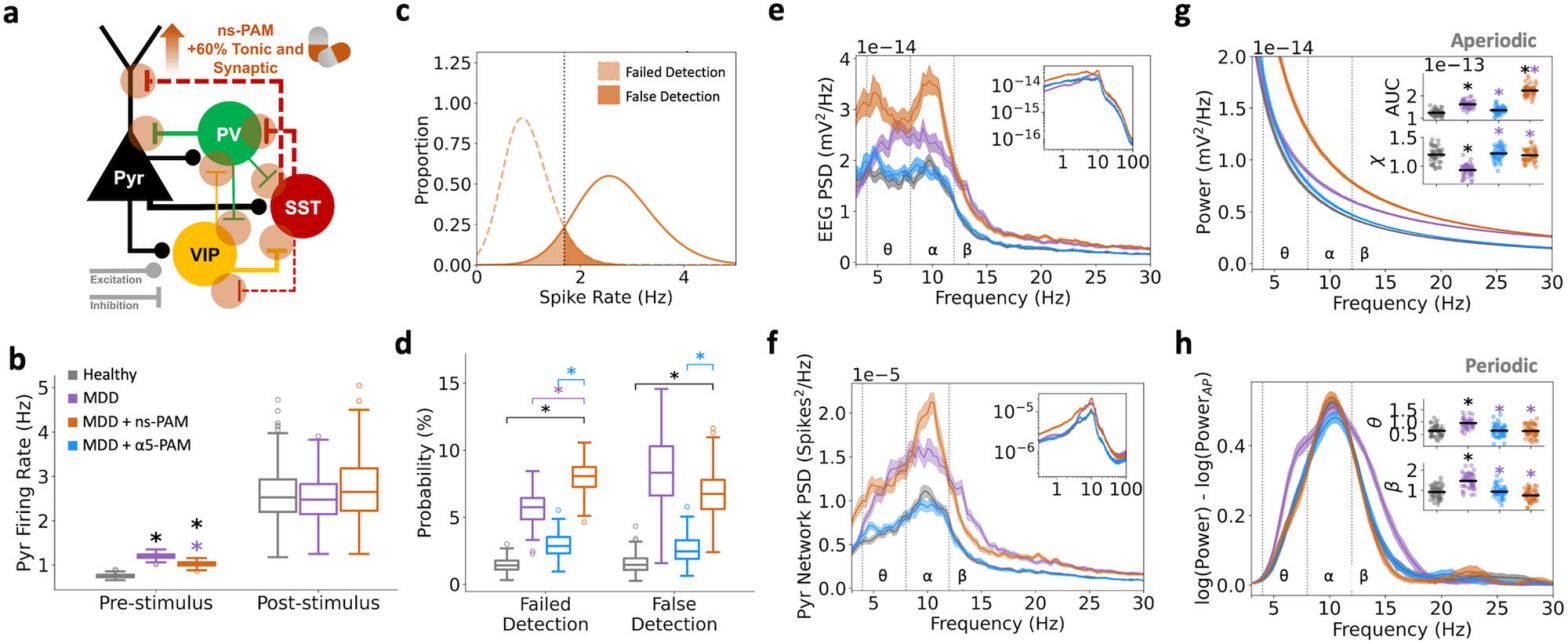
In silico application of non-selective PAM fails to recover microcircuit function and EEG. **a** Schematic of non-selective PAM (ns-PAM) effects on the model microcircuit. **b** Pre- and post-stimulus Pyr neuron firing rates in the different conditions. **c** Distributions of pre- and post-stimulus firing rates. **d** Probability of failed detection and false detection with non-selective PAM was worsened and unchanged, respectively, compared to the depression condition (MDD). **e** EEG PSD, bootstrapped mean, and 95% confidence intervals. Inset: EEG PSD in log scale. **f** Spikes PSD of Pyr neurons, bootstrapped mean, and 95% confidence intervals. Inset: spikes PSD in log scale. **g** Fitted aperiodic components of the EEG PSD for each condition. Upper inset: broadband (3–30 Hz) area under the curve (AUC). Lower inset: exponent (χ). **h** Fitted periodic component of the EEG PSD for each condition. Inset plots: integral of the power spectral density in the theta (θ) and beta (β) frequency ranges. All asterisks denote significant paired *t*-tests (*p* < 0.05) with effect sizes greater than 1, when compared to healthy (black asterisks) or depression (MDD; purple asterisks). *n* = 200 randomized microcircuit simulations per condition for panels **a**–**d**. *n* = 50 randomized microcircuit simulations per condition for panels **e**–**h**. For all box-and-whisker plots, the boxes show the interquartile range, the middle lines show the medians, the whiskers extend to all data within 1.5x the IQR, and dots show datapoints outside of the whisker range.

We next compared the efficacy and EEG biomarkers of simulated α5-PAM with those of non-selective PAM, e.g. corresponding to a non-selective benzodiazepine (boosting a broad range of GABA_A_ subunits), by simulating a 60% increase in tonic and synaptic inhibition in all of the microcircuit inhibitory connections (Fig. 7a). Though simulated non-selective PAM reduced baseline microcircuit spike rates, it did not restore spike rates sufficiently back to healthy levels (Fig. 7b; non-selective PAM compared to healthy: 1.02 ± 0.06 Hz, paired-sample *t*-test, *p* = 1.06e−178, Cohen’s *d* = 5.5). Accordingly, simulated non-selective PAM did not improve false detection rate (Fig. 7c-d; non-selective PAM: 6.78 ± 1.73%, healthy: 1.54 ± 0.63%, paired-sample t-test, *p* = 2.58e−103, Cohen’s *d* = 4.0) and even worsened the failed detection rate (non-selective PAM: 7.99 ± 1.17%, healthy: 1.45 ± 0.47%, paired-sample t-test, *p* = 1.61e−158, Cohen’s *d* = 7.3).

We analyzed the effects of non-selective PAM on simulated EEG power spectral density profile and found increased power in all frequency bands (Fig. 7e). Increased broadband power was similarly seen in the PSD of the Pyr neuron spiking (Fig. 7f). When decomposed into aperiodic and periodic components, we found that non-selective PAM caused a broadband upward shift in the aperiodic component (Fig. 7g, healthy: 1.24 × 10^−13^ ± 1.29 × 10^−14^ mV^2^; non-selective PAM: 2.24 × 10^−13^ ± 2.35 × 10^−14^ mV^2^; paired-sample t-test, *p* = 2.15e−33, Cohen’s *d* = 5.2), but recovered the aperiodic exponent parameter (Fig. 7g inset, healthy: 1.20 ± 0.13 mV^2^ Hz^−1^; non-selective PAM: 1.19 ± 0.10 mV^2^ Hz^−1^; Cohen’s *d* = −0.1) as well as the periodic theta power (Fig. 7h inset, healthy: 0.64 ± 0.20 mV^2^; non-selective PAM: 0.63 ± 0.23 mV^2^; Cohen’s *d* = −0.01) and low-beta power (Fig. 7h inset, healthy: 0.92 ± 0.30 mV^2^; non-selective PAM: 0.76 ± 0.27 mV^2^; Cohen’s *d* = −0.5).

## Discussion

In this work, we tested in silico the effects of α5-PAM in detailed human cortical microcircuit models of depression and found that several indicators quantifying microcircuit dynamics, function, and EEG profile were brought back to healthy levels. We showed that the functional recovery, measured as failed and false detection, was on the same order of magnitude as the pro-cognitive effects measured in chronically stressed mice^31^. We further identified EEG biomarkers of different dose effects, highlighting recovery of EEG power in theta and beta frequencies, as well as broadband recovery. This mechanistic demonstration of α5-PAM efficacy on human cortical microcircuits could serve to guide pre-clinical studies, de-risk and facilitate translation to human clinical use, and provide candidate biomarkers in non-invasive brain signals for monitoring drug efficacy. In particular, our in-silico biomarker candidates can be tested in improving patient stratification and treatment outcome prediction, by identifying those that have the relevant depression EEG profile and that could benefit from being administered α5-PAM. Our in silico approach thus complements experimental human and rodent research in giving a snapshot of how α5-PAMs will impact human brain activity in vivo, which is not data that is typically accessible clinically, especially in the context of novel drug testing.

Our results demonstrated that α5-PAM could directly recover function and resting state EEG features associated with a loss of SST interneuron inhibition in depression^48,49^, despite only boosting SST→Pyr synapses and not SST → PV and SST → VIP synapses, which were also reduced in our depression models. α5-PAM also recovered SST and PV interneurons rates, and partially VIP interneuron rates to healthy levels. This is likely because the loss of SST interneuron inhibition in depression has a direct and thus much larger impact on Pyr neurons than the indirect disinhibitory effect of reduced SST interneuron inhibition onto interneurons. In agreement with this rationale, we demonstrated that the use of a non-selective PAM failed to recover circuit activity to healthy levels, likely due to an indiscriminatory boosting of inhibition throughout the circuit rather than localized to the SST→Pyr connections. Similarly, while non-selective PAM dampened the elevated spike rates in depression to some extent, in line with previous studies during application of different non-selective benzodiazepines in rodent cortical cultures^50^, the effects were small compared to α5-PAM effects, possibly as a result of the non-selective PAM boosting of all inhibitory connections in the microcircuit. We used a simple model of the effects of non-selective PAMs as a general reduction in inhibition since there is insufficient data to allow modeling of the effects of any specific non-selective drug compound on human neurons.

Recovery of theta, alpha, and lower beta frequency band power as a result of α5-PAM administration are relevant to depression diagnosis and severity because studies have shown that there are power increases in these specific bands in depression^49,51–54^. These potential biomarkers could thus be used as indicators of treatment response^55–57^. We note that although beta periodic peaks and beta oscillatory events were observed in our models, the average time-collapsed PSD did not show apparent beta peaks. This is primarily due to the averaging across different microcircuits, as well as the smaller power in beta compared to the alpha tail. Including other beta-promoting mechanisms, such as the dynamical contributions from layer 5 cortical circuits could increase the power of beta oscillations in the model^58,59^. Analyses in the time-frequency domain further revealed that the increased power in depression involved increases in oscillatory event wave amplitudes across all frequency bands, which was recovered to a healthy level with α5-PAM. Similar to proposals from previous modeling^60^ and experimental^61^ studies, this approach offers additional biomarkers that are at a higher temporal resolution than the PSD. However, we note that using an oscillatory event-based approach does not dissociate between periodic and aperiodic elements, and thus the aperiodic/periodic decomposition of the power spectrum was equally useful.

Our results demonstrate mechanistically that α5-PAM can directly recover resting state aperiodic and periodic power spectral biomarkers (i.e. corresponding to measures of asynchronous and synchronous microcircuit dynamics, respectively) associated with a loss of SST interneuron inhibition in models of depression^49^. Across all functional metrics and types of EEG biomarkers, simulated dose effects were linear, in line with the gradual α5-PAM dose-effects seen behaviourally in rodents^31^. In comparison, the simulated non-selective PAM did not recover the power spectral profile. This is in agreement with previous studies showing that non-selective benzodiazepines and benzodiazepines selective for α1 subunits have been associated instead with increases in EEG delta and beta rhythms in humans during resting-state^62–64^, and with a slowing of the peak frequency over time^62^. These findings are consistent with the poor effectiveness of non-selective PAM at treating depression, as suggested by previous clinical studies^65^.

We constrained the models with the effect of 3 μM of the α5-PAM compound, GL-II-73, which was in the previously demonstrated range for selectively targeting α5 subunit receptors compared to higher concentrations that more strongly target α1, α2, and α3 subunits^26,31^. We assumed that the α5-PAM effect on tonic inhibition conductance, which we estimated from the human neuronal recordings, would also occur with synaptic conductance of SST→Pyr connections because of the localization of α5 subunits in those synapses^20–23^. The proportion of α5-PAM effects on synaptic or tonic inhibition would depend on trafficking of α5 subunits to extrasynaptic and synaptic locations, which is highly dynamic and activity-dependent^21,28^. Further data of α5-PAM effect on synaptic vs tonic inhibition could thus refine the models and increase the accuracy of their predictions. Since α5 subunits in human cortex are mainly expressed in Pyr neurons, with negligible expression in interneurons^24^, we have taken a simplified modeling approach whereby α5-PAM modulated only the inhibition onto apical dendrites in Pyr neurons. As more data becomes available about the expression and distribution of α5 subunits in different cell types, our models can be refined to better mimic the dose-dependent effects of α5-PAMs on the microcircuits. In addition, we relied on experimental data of α5-PAM modulation of Pyr neurons in temporal lobe tissue and thus considered the models as prototypical microcircuits, yielding general predicted effects without regional specificity. Future studies characterizing the modulation in other areas, especially those relevant to depression, can refine our predictions.

We tested α5-PAM effect on cortical function using signal detection metrics that relate to deficits in depression^66–68^, possibly due to increased noise in cortical processing as a result of reduced SST interneuron inhibition^48^. As in previous work^48^, we did not observe any significant impacts on post-stimulus firing rates, and we assumed that deficits involving SST interneurons are imposed through increased baseline rates. Comparing population response firing rates to baseline firing rates is a commonly used method for studying fundamental properties underlying cognition^69^, as this computation is expected to be made by downstream neurons to differentiate incoming information. In depression, the reduced SST interneuron inhibition increased microcircuit activity and thus noise levels in signal detection, as has been posited in depression literature^9^. Administration of α5-PAM and characterization of its effects on cognition has previously only been done in rodents^29,31^. Whereas both sensory and memory impairments are thought to be linked to increased noise in cortical processing, although in different corresponding cortical regions^8,9^, future simulation studies will benefit from testing α5-PAM effects on other, more complex, cognitive functions than those we have modeled.

Our simulations focused on the modelling the effects of GL-II-73 α5-PAMs, which are prepared from the SH-053-2’F-R-CH3 compound^31^. Modelling the effects of SH-053-2’F-R-CH3 itself and other related compounds would first require electrophysiological characterization on human Pyr neurons, but would likely generate qualitatively similar effects, due to the higher binding affinities for α5-GABA_A_ receptor subunits, with possible different drug efficacy depending on α5 selectivity. We also note that negative allosteric modulators of α5 (α5-NAM) have also been proposed to have therapeutic potential^28^. Although α5-NAM could worsen our depression cortical microcircuits due to their opposite effect on SST interneuron inhibition, determining the net effect on the microcircuit will require simulations constrained by similar data of electrophysiological characterization such as single-cell effect, as we have done for α5-PAM. We also note that the reference dose of GL-II-73 that we simulated (3 µM) roughly equates to a 1.16 mg kg^-1^ dose. In rodents, this dose is within the range for optimally activating α5-GABA_A_ receptors without substantial activation of other subunit receptors, thus yielding anxiolytic, antidepressant, and pro-cognitive effects without unwanted effects such as sedation^31^. Though these experiments support our chosen reference dose, we note that differences between rodents and humans, as well as the particular drug administration method, may yield different acute brain concentrations. Therefore, further experiments will be required to verify whether the same dose is sufficient for humans, or whether some adjustments are needed to yield the same effective dose in human brain tissue.

As in previous studies, we used models of human cortical L2/3 microcircuits to study effects on cortical microcircuit function^48^ and EEG signal^49^. These serve as prototypical models of human cortical microcircuitry, as supported by their ability to reproduce key aspects of human resting-state EEG recordings^49^, due to the use of realistic human neuronal morphologies, human synaptic properties, and the key neuron types in the microcircuit. The inclusion of morphologies enabled the estimation of the resulting EEG and captured features of the cortical microcircuit relevant to the depression model such as targeted SST inhibition of apical dendrites. Further support for the predictive power of the L2/3 microcircuits stems from L2/3 being closest to the EEG electrode and thus its apical pyramidal dipoles are major contributors to EEG signals^70^. Future expanded models that include layers 4 and 5, which also contribute substantially to EEG signals, will refine our estimated EEG biomarkers of α5-PAM effects, by including the long superficially-projecting apical dendrites from deeper layer pyramidal neurons, additional complexities of interlaminar oscillatory communication^71^, and oscillatory dynamics present in deeper cortical layers^58,59^. The inclusion of layer 1 interneuron populations, of which there is increasing human data available^72,73^, could also refine our models, given that interneurons in this layer also provide inhibition to Pyr neuron apical dendrites and could thus experience a boost using α5-PAMs. Addition of these layers and cell type populations will help refine our models, and allow closer approximations to real data, (e.g., such as in Dura-Bernal et al.^74^). Although we simulated L2/3 microcircuits ~6–7 times smaller in terms of cell numbers than the true size (for computational efficiency), the down-sampling involved proportional changes in both excitatory and inhibitory neurons, which maintained the overall excitatory–inhibitory balance of the network. We do, however, expect our biomarkers will largely hold, so that any refinements will rather serve to provide additional biomarker candidates such as phase-amplitude coupling between different frequency bands originating from different layers^71^.

Whereas we used models of depression microcircuits with reduced SST interneuron inhibition that were constrained with expression data from depression patients^48^, other mechanisms of depression include morphological atrophy and reduced spine density^26,75^. However, we note that chronic α5-PAM exposure was shown to be effective in directly recovering these morphological features^26,76^, therefore the translation of α5-PAM effects on these depression mechanisms to humans would be more trivial. As well, while reduced SST expression was observed in the human subgenual anterior cingulate cortex^10,77^, our models were developed using data from the human middle temporal cortex, which is the main source of human neuronal and synaptic data^48^. We, therefore, considered our models to be of prototypical cortical microcircuits, but when human neuronal data and models of prefrontal cortical microcircuits become available^78^, they can be used to enable relevant region specificity. Whereas cortical layer SST expression loss in depression was more severe in women than in men^10,77^, imaging of SST expression selectively within SST interneurons showed strong reductions in both men and women in depression^10^. Thus, it remains unclear whether the sex difference in bulk SST expression would translate to functional differences in loss of SST interneuron inhibition in depression, and thus difference in predicted dose of α5-PAM. As further data on inhibitory signaling via SST becomes available, computational models of depression should also consider the direct impact of altered inhibitory SST receptor signaling^18^ rather than using only altered GABAergic signaling by proxy.

Altogether, our results provide the first demonstration that α5-PAM intervention in the context of human depression could recover the level of SST interneuron inhibition, SNR of cortical processing and fundamental cortical function exemplified by signal detection. Our results thus suggest that α5-PAM intervention could have a therapeutic effect on cognition in human depression similar to that demonstrated previously in rodents. Our study also presents a first in-silico testing of pharmacology systematically on detailed models of human cortical microcircuits, which we hope will also be of service in future efforts.

## Methods

### Electrophysiology data

We used whole-cell voltage-clamp recordings of tonic inhibition current in the presence of GABA only, and in the presence of α5-PAM + GABA, in human cortical L2/3 Pyr neurons (10 cells: 9 cells from 3 male subjects, 1 cell from 1 female subject) from patients undergoing a selective amygdalohippocampectomy^79^. As described in previous work^80^, the resected cortical tissue was considered healthy as it was located outside of the site of epileptogenesis. Written informed consent was obtained from all participants, in accordance with the Declaration of Helsinki and the University Health Network Research Ethics board. All ethical regulations relevant to human research participants were followed.

The data was collected using surgery resection, solutions, tissue preparation, and recording equipment described previously^48,80,81^. Neocortical tissue resected during anterior temporal lobectomy was immediately submerged in ice-cold (~4°C) cutting solution and transferred to a recording chamber within 20 minutes. After sectioning the tissue, the slices were incubated for 30 min at 34 °C in standard artificial cerebrospinal fluid (aCSF) (in mM): NaCl 123, KCl 4, CaCl_2_.2H_2_O 1.5, MgSO_4_.7H_2_O 1.3, NaHCO_3_ 26, NaH_2_PO_4_.H_2_O 1.2, and D-glucose 10, pH 7.40 and bubbled with carbogen gas (95% O_2_–5% CO_2_) and had an osmolarity of 300–305 mOsm.

For recordings, slices were transferred to a recording chamber mounted on a fixed-stage upright microscope (Axioskop 2 FS MOT; Carl Zeiss, Germany). Slices were continually perfused at 4 ml min^-1^ with standard aCSF at 32–34 ^o^C. Whole-cell patch-clamp recordings were obtained using a Multiclamp 700 A amplifier and pClamp 10.6 data acquisition software (Axon instruments, Molecular Devices, USA). Subsequently, electrical signals were digitized at 20 kHz using a 14140 A digitizer. For voltage-clamp recordings of tonic current, low-resistance patch pipettes (2–4 MΩ) were filled with a CsCl-based solution containing (in mM) 140 CsCl, 10 EGTA, 10 Hepes, 2 MgCl_2_, 2 Na_2_ATP, 0.3 GTP, and 5 QX314 adjusted to pH 7.3 with CsOH. The junction potential was calculated to be 4.3 mV and the holding potential was −74.3 mV after junction potential correction. As in previous studies^23,82^, in this configuration, 5 μM GABA, 25 μM AP5, 10 μM CNQX, and 10 μM CGP-35348 were first applied to generate larger GABA-dependent currents and assess tonic inhibition currents while also blocking AMPA, NMDA, and GABA_B_ mediated currents. 3 μM of α5-PAM compound GL-II-73^31^ was then applied to assess tonic inhibition current in the presence of α5-PAM, followed by 50 μM of picrotoxin to block GABA_A_ mediated currents and assess endogenous current output during voltage-clamp recordings without any synaptic activity.

### Human cortical microcircuit models in health and depression

We used morphologically- and biophysically-detailed models of human L2/3 cortical microcircuits in health and depression described previously^48^. Briefly, these microcircuit models were comprised of 1000 neurons (80% Pyr, 5% SST, 7% PV, and 8% VIP) distributed across a 500 x 500 x 950 µm^3^ volume and simulated using NEURON 7.7^83^ and LFPy 2.0.2 (Python 3.7.6)^84^. Microcircuit simulations were run on SciNet parallel computing^85^, using 400 nodes, with a runtime of ~10 mins per 4.5 s microcircuit simulation. Our models^48^ were constrained with human data where available, primarily middle temporal gyrus (electrophysiology of different neuron types, morphologies, Pyr-Pyr, SST-Pyr, Pyr-SST, PV-Pyr, and Pyr-PV synaptic connections, in-vivo Pyr baseline firing rate, and Pyr tonic inhibition in health and α5-PAM) and anterior cingulate cortex (SST gene expression in health and depression). Otherwise, rodent somatosensory cortex data was used (other synaptic connections not listed above, in-vivo SST, PV, and VIP interneuron baseline firing rates, and in-vivo response firing rates across neuron types). The neuronal morphology reconstructions of the multi-compartment models were obtained from the Allen Cell Types database^86^, and the models were fitted using multi-objective optimization^87,88^ with either single-cell data from the Allen Brain Institute (putative PV, SST, and VIP inhibitory neuron fits)^86^ or population Pyr neuron data from the Krembil Brain Institute^80,81^. Synaptic parameters in these models were fit to human data where possible^12,39,41,89^ and to curated rodent data otherwise^90^. In terms of connections onto Pyr neurons which had two types of dendritic trees (basal and apical), the Pyr→Pyr excitatory synapses were placed on both basal and apical dendritic compartments, the PV→Pyr inhibitory connections were placed on basal dendritic compartments, the SST→Pyr inhibitory connections were placed on apical dendritic compartments. Depression microcircuits were modelled by reducing the conductance of SST interneuron synaptic and tonic inhibition onto all cell types by 40%^48^. For Pyr neurons in the depression model, tonic inhibition conductance was reduced by 40% on only apical dendritic compartments. For each interneuron type in the depression model, the contributions of SST interneurons to tonic inhibition were estimated and this contribution was reduced by 40%. Randomizing the circuit comprised of sampling synaptic connections, neuron positions in space, background noise input, and spike timing of thalamic inputs (see section below). Full model details are available in Yao et al.^48^, and the models are openly accessible online: 10.5281/zenodo.5771000^91^.

### Modelling microcircuit baseline and response activity

As in previous work^48^, the microcircuit generated spike rates at baseline and during response in line with the different neuron types in vivo^14,92,93^, and each neuron received random background excitatory inputs using Ornstein-Uhlenbeck (OU) point processes^94^. Briefly, independent OU point processes were placed at the midway points along the length of each dendritic arbor, and for Pyr neuron models, we placed 5 additional OU processes along the apical dendrites (i.e., at 10%, 30%, 50%, 70%, 90% of the apical length). We set the inhibitory OU conductance to 0. For the mean and standard deviation of the excitatory OU conductance, we set these values to be equivalent to each other and scaled them to increase with relative distance from soma (ranging from 0 to 1), starting from an inputted magnitude value ($g$), as follows: ${\bar{g}}_{{OU}},{\sigma }_{{OU}}=g\times \exp ({X}_{{relative}})$.

As in Yao et al.^48^, we reproduced response rates using excitatory AMPA/NMDA synapses with the same synaptic dynamics and number of contacts as the cortical excitatory synapses. 55 Pyr neurons were stimulated in the basal dendrites, with 2–4 ms delay post-stimulus and a conductance of 4 nS. 35 PV interneurons were stimulated with a delay of 2–2.5 ms and a conductance of 2 nS. VIP interneurons were stimulated in two groups and phases: early (65 VIP interneurons, delay = 0.5–4.5 ms, conductance = 2.8 nS) and late (80 VIP interneurons, delay = 7–12 ms, conductance = 2.2 nS). Average post-stimulus rates were calculated over the 5–55 ms window after stimulus onset. For simulations where we tested excitatory stimulation of Pyr neuron apical dendrites instead of basal dendrites, 85 Pyr neurons were stimulated by synapses spread randomly across the apical dendrites. In these cases, all other stimulus parameters remained the same as in the basal stimulation case.

### Tonic inhibition models

As in Yao et al.^48^, we used a model for outwardly rectifying tonic inhibition^95^ as well as the tonic inhibition conductance values that had previously been fitted to capture the current magnitudes recorded in human L2/3 Pyr neurons in the presence of GABA only (see electrophysiology methods above and Yao et al.^48^). We simulated the experimental conditions by setting the inhibitory chloride reversal potential to -5 mV (consistent with the experimental solutions), setting the holding potential to -75 mV in voltage-clamp mode, and tuning the tonic inhibition conductance on all Pyr neuron somatic and dendritic compartments to reproduce the target experimental tonic inhibition current amplitude (resulting in *G*_*tonic*_ = 0.938 mS cm^−2^). The same *G*_*tonic*_ value was used for the interneurons since the total tonic inhibition current recorded in interneurons is similar to that of Pyr neurons after correcting for cell capacitance^25^.

### α5-PAM models

We estimated the *G*_*tonic*_ modulation on Pyr neuron apical dendrites during application of α5-PAM (resulting in *G*_*tonic*_ = 1.498 mS cm^−2^) using the experimental tonic inhibition currents as target magnitudes, and the simulation settings as described for tonic inhibition models above. All simulated current magnitudes were calculated relative to the endogenous current magnitude generated in the condition where *G*_*tonic*_ is set to 0 mS cm^−2^, corresponding to the picrotoxin condition in the experimental methodology. We then applied the estimated α5-PAM modulation effect on both tonic and synaptic inhibition, corresponding to *G*_*tonic*_ and SST→Pyr synaptic conductance (*G*_*SST→Pyr*_) in the microcircuit models. We simulated different doses of α5-PAM ranging from 25% to 150% of the estimated modulation effect of the experimental reference dose.

### Non-selective PAM models

We modeled the action of non-selective GABA_A_ receptor PAM (i.e. benzodiazepines broadly non-selective for α1-, α2-, α3- and α5 subunit-containing GABA_A_ receptors) by applying the same magnitude of estimated α5-PAM modulation, but to all synaptic and tonic inhibition connections in the microcircuit.

### Failed/false signal detection rates

We computed error rates in stimulus processing with our microcircuit models as in previous work^48^, by first fitting the pre-stimulus firing rate distributions (computed using a 50 ms sliding window, sliding in 1 ms intervals, over a 3 s pre-stimulus period) to skewed normal distributions for each of the 200 randomized microcircuits (n = 2951 windows × 200 microcircuits pre-stimulus). We then fitted the post-stimulus firing rate distribution (in the 5–55 ms period post-stimulus) across all the 200 randomized microcircuits (*n* = 200 windows). 200 randomized microcircuit simulations were necessary in this analysis to adequately estimate the post-stimulus firing rate distributions. The intersection point between pre-stimulus distribution and the post-stimulus distribution was chosen to be the stimulus detection threshold, in line with optimal decision theory^96^. Probability of false detections was computed as the integral of the pre-stimulus distribution above the detection threshold divided by the integral of the entire pre-stimulus distribution. The probability of failed detections was computed as the integral of the post-stimulus distribution under the detection threshold divided by the integral of the entire post-stimulus distribution.

### Simulated microcircuit EEG and power spectral analyses

We simulated dipole moments and corresponding EEG time series data generated by our microcircuit models (25 s duration per simulation) using the same methodologies as in previous work^49,84^. Because these simulations were of long duration and the saved output dipole moments were considerably larger file sizes than the saved spike trains, we limited the number of randomized microcircuit simulations to 50. Specifically, we used a four-sphere volume conductor model (representing grey matter, cerebrospinal fluid, skull, and scalp with radii of 79 mm, 80 mm, 85 mm, and 90 mm, respectively) that assumed homogeneous, isotropic, and linear (frequency-independent) conductivity. The conductivity for each sphere was 0.047 S m^−1^, 1.71 S m^−1^, 0.02 S m^−1^, and 0.41 S m^−1^, respectively^49,97^. We computed EEG power spectral density using Welch’s method^98^ from the SciPy python module, and quantified power spectral features by computing the integral of the power spectral densities for theta (4–8 Hz), alpha (8–12 Hz), and lower beta (12–21 Hz) range frequencies.

As in previous work^49^, we decomposed the EEG power spectral densities (in the 3–30 Hz range) into aperiodic and periodic components using an algorithmic parameterization method^99^. The aperiodic component was a 1/f function parameterized by vertical offset and exponent parameters. As an additional quantification metric, we computed the integral of the broadband (3–30 Hz) frequency range in the aperiodic component (or area-under the curve, AUC). Overlying the aperiodic component, we fitted the periodic oscillatory component with up to 3 Gaussian peaks which were defined by center frequency, bandwidth (min: 2 Hz, max: 6 Hz), and power magnitude (relative peak threshold: 2, minimum peak height: 0). We quantified periodic features by computing the integral of the periodic component for theta, alpha and lower beta range frequencies.

We also computed the spiking power spectral density of Pyr neurons by converting the spike times into binary spike train vectors, which we then summed across all Pyr neurons. Power spectral density was then computed from the summed spike train vectors using Welch’s method^48,98,100^. For both Pyr neuron spiking and EEG, we computed power spectral density with nperseg = 140,000 sampling points, which was equivalent to 3.5 s time windows. For spiking and EEG power spectral density vectors, as well as the aperiodic and periodic vectors, across random seeds we computed the bootstrapped (100 iterations) means and 95% confidence intervals for each frequency.

### Oscillatory event analysis

To assess the impact of depression and recovery via α5-PAM on individual oscillatory events in the EEG signals, we performed a wavelet-based spectrogram analysis using the toolbox OEvents^74,101^ in python. We used the following parameters: medthresh = 4.0 (median threshold); sampr = 40,000 Hz (sampling rate); winsz = 24 seconds (window size), freqmin = 1 Hz (minimum frequency); freqmax = 100 Hz (maximum frequency); freqstep = 0.5 Hz (frequency step); overlapth = 0.5 (overlapping bounding box threshold). Metrics were computed and averaged across 50 randomized circuits for each condition. We note that the OEvents toolbox classifies events using frequency band ranges that are slightly different from the ranges we have used throughout the rest of our manuscript: δ (0.5–4 Hz), θ (4–9 Hz), α (9–15 Hz), β (15–29 Hz), low γ (30–40 Hz), γ (40–80 Hz). Events were classified into bands based on the frequency at which peak power occurred.

### Statistics and reproducibility

Unless otherwise indicated, for group comparisons we used two-sided paired-sample t-tests. For linear correlations, we used two-sided Pearson correlations. Cohen’s *d* was calculated as follows:

$${Cohe}{n}^{{\prime} }{sd}=\frac{\bar{x}-\bar{y}}{\sqrt{\frac{({N}_{x}-1)\times {{\sigma }_{x}}^{2}+({N}_{y}-1)\times {{\sigma }_{y}}^{2}}{{N}_{x}+{N}_{y}-2}}}$$

Numbers of simulations, simulation duration, as well as rationale for these numbers, for each test, are stated in the appropriate Methods sections.

### Reporting summary

Further information on research design is available in the Nature Portfolio Reporting Summary linked to this article.

### Supplementary information

Reporting Summary

## Data Availability

All simulations and figures can be replicated using the custom code and numerical data in the repository provided in the code availability section.

## Code availability

All original code has been deposited on Github and is publicly available as of the date of publication (https://github.com/agmccrei/HumanL23Circuit_a5PAM_AGM2023)^102^.
