# Supplementary material for: In-silico testing of new pharmacology for restoring inhibition and human cortical function in depression
Source: Commun Biol. 2024 Feb 23;7:225. doi: 10.1038/s42003-024-05907-1 (PMC10891083; doi:10.1038/s42003-024-05907-1)
Supplement: Supplementary file 1 — Reporting Summary [file 42003_2024_5907_MOESM1_ESM.pdf]

nature portfolio

Corresponding author(s): Etay Hay

Last updated by author(s): Jan 12, 2024

Reporting Summary

Nature Portfolio wishes to improve the reproducibility of the work that we publish. This form provides structure for consistency and transparency in reporting. For further information on Nature Portfolio policies, see our Editorial Policies and the Editorial Policy Checklist.

Statistics

For all statistical analyses, confirm that the following items are present in the figure legend, table legend, main text, or Methods section.

n/a	Confirmed	
[ ]	[x]	The exact sample size (*n*) for each experimental group/condition, given as a discrete number and unit of measurement
[ ]	[x]	A statement on whether measurements were taken from distinct samples or whether the same sample was measured repeatedly
[ ]	[x]	The statistical test(s) used AND whether they are one- or two-sided<br>*Only common tests should be described solely by name; describe more complex techniques in the Methods section.*
[ ]	[x]	A description of all covariates tested
[x]	[ ]	A description of any assumptions or corrections, such as tests of normality and adjustment for multiple comparisons
[ ]	[x]	A full description of the statistical parameters including central tendency (e.g. means) or other basic estimates (e.g. regression coefficient) AND variation (e.g. standard deviation) or associated estimates of uncertainty (e.g. confidence intervals)
[ ]	[x]	For null hypothesis testing, the test statistic (e.g. *F*, *t*, *r*) with confidence intervals, effect sizes, degrees of freedom and *P* value noted<br>*Give P values as exact values whenever suitable.*
[x]	[ ]	For Bayesian analysis, information on the choice of priors and Markov chain Monte Carlo settings
[x]	[ ]	For hierarchical and complex designs, identification of the appropriate level for tests and full reporting of outcomes
[ ]	[x]	Estimates of effect sizes (e.g. Cohen's *d*, Pearson's *r*), indicating how they were calculated

Our web collection on statistics for biologists contains articles on many of the points above.

Software and code

Policy information about availability of computer code

Data collection	NEURON 7.7 (simulation software), LFPy 2.0.2 (simulation software), Yao et al, 2022 (https://doi.org/10.5281/zenodo.5771000; simulation model), custom code for simulation conditions (private repository - https://github.com/agmccrei/HumanL23Circuit_a5PAM_AGM2023 - will be made public upon publication)
Data analysis	Custom code (private repository - https://github.com/agmccrei/HumanL23Circuit_a5PAM_AGM2023 - will be made public upon publication).

For manuscripts utilizing custom algorithms or software that are central to the research but not yet described in published literature, software must be made available to editors and reviewers. We strongly encourage code deposition in a community repository (e.g. GitHub). See the Nature Portfolio guidelines for submitting code & software for further information.

Data

Policy information about availability of data

All manuscripts must include a data availability statement. This statement should provide the following information, where applicable:

- Accession codes, unique identifiers, or web links for publicly available datasets
- A description of any restrictions on data availability
- For clinical datasets or third party data, please ensure that the statement adheres to our policy

All simulated data can be replicated using the custom code in the available repository provided in the code availability section.

Research involving human participants, their data, or biological material

Policy information about studies with human participants or human data. See also policy information about sex, gender (identity/presentation), and sexual orientation and race, ethnicity and racism.

Reporting on sex and gender	Due to limited data availability, neither sex nor gender effects could be assessed.
Reporting on race, ethnicity, or other socially relevant groupings	Due to limited data availability, effects of these variables were not assessed.
Population characteristics	Due to limited data availability, effects of these variables were not assessed.
Recruitment	All experimental data was obtained from patients undergoing a selective amygdalohippocampectomy, however the resected cortical tissue was considered healthy as it was located outside of the site of epileptogenesis
Ethics oversight	University Health Network

Note that full information on the approval of the study protocol must also be provided in the manuscript.

Field-specific reporting

Please select the one below that is the best fit for your research. If you are not sure, read the appropriate sections before making your selection.

☒ Life sciences ☐ Behavioural & social sciences ☐ Ecological, evolutionary & environmental sciences

For a reference copy of the document with all sections, see nature.com/documents/nr-reporting-summary-flat.pdf

Life sciences study design

All studies must disclose on these points even when the disclosure is negative.

Sample size	Sample size was limited by the number of patients undergoing a selective amygdalohippocampectomy.
Data exclusions	No data was excluded.
Replication	Experimental results were not replicated due to limited data availability.
Randomization	Randomization was not relevant to this study. Tissue was collected from all patients and underwent the same experimental procedure.
Blinding	Blinding was not relevant to this study. Experimental tests assessed electrophysiological changes during drug application.

Reporting for specific materials, systems and methods

We require information from authors about some types of materials, experimental systems and methods used in many studies. Here, indicate whether each material, system or method listed is relevant to your study. If you are not sure if a list item applies to your research, read the appropriate section before selecting a response.

Materials & experimental systems

n/a	Involved in the study	
☒	☐	Antibodies
☒	☐	Eukaryotic cell lines
☒	☐	Palaeontology and archaeology
☒	☐	Animals and other organisms
☒	☐	Clinical data
☒	☐	Dual use research of concern
☒	☐	Plants

Methods

n/a	Involved in the study	
☒	☐	ChIP-seq
☒	☐	Flow cytometry
☒	☐	MRI-based neuroimaging

Plants

Seed stocks	N/A
Novel plant genotypes	N/A
Authentication	N/A
